# Representing life as opposed to being: the bio-objectification process of the *HeLa* cells and its relation to personalized medicine

**DOI:** 10.3325/cmj.2013.54.397

**Published:** 2013-08

**Authors:** Anna Lydia Svalastog, Lucia Martinelli

**Affiliations:** 1 Centre for Research Ethics and Bioethics, Uppsala University, Uppsala, Sweden *anna-lydia.svalastog@crb.uu.se*; 2 Museo delle Scienze – MUSE, Trento, Italy *lucia.martinelli@muse.it*

## Abstract

The immortal *HeLa* cells case is an intriguing example of bio-objectification processes with great scientific, social, and symbolic impacts. These cells generate questions about representation, significance, and value of the exceptional, variety, individuality, and property. Of frightening (a lethal cancer) and emarginated (a black, poor woman) origins, with their ability to “contaminate” cultures and to “spread” into spaces for becoming of extraordinary value for human knowledge, well-being, and economy advancements, *HeLa* cells have represented humanity, and emphasized the importance of individual as a core concept of the personalized medicine. Starting from the process leading from *HeLa* “cells” to *HeLa* “bio-objects,” we focus on their importance as high quality bio-specimen. We discuss the tension between phenomenological characteristic of fundamental biological research and the variety of material and methodologies in epidemiology and personalized medicine. The emerging methodologies and societal changes reflect present EU policies and lead toward a new paradigm of science.

Current biotechnology is characterized by its capacity to generate biological processes and analyze large amounts of information supported by the development of advanced equipments. Biotechnology has opened up unique potentials for producing new objects by manipulating and transgressing boundaries between domains that were formerly understood as incompatible, or by creating completely new materials. These new entities are named bio-objects (1), and they are defined as biology innovations produced through processes continuously negotiated in the intersection of science, politics, and society (2). In this definition, bio-objects are approached as temporary categories that are produced in an ongoing bio-objectification process, aiming at controlling life in specific time and space.

When mentioning “bio-objects” as novel biological entities, a special place has to be assigned to the *HeLa* cells for their capability to challenge conventional natural, cultural, scientific, and institutional classifications (bio-objects) and to generate controversy due to their potential challenging of established order and practices (bio-objectification). Thus, under the lenses of bio-object and bio-objectification concepts, various remarkable features may be attributed to *HeLa* cells and to the controversial bio-ethical arguments their establishment and use still generate today, six decades later.

## The *HeLa* cells

*HeLa* cells are an immortalized line established in the late 1950s, from a rare cervix adenocarcinoma of a young woman. They were named after her: Henrietta Lacks. These cells became, and still are, one of – if not the most important one – laboratory model of modern cell biology research since its first establishment in 1959. They were applied to study crucial biological processes of healthy and pathological systems, the functions of genes, and the development of pioneer “omics” approaches, as also proved by the over 60 000 publications produced, according to MEDLINE database (3). They were also necessary for relevant research that was awarded with two Nobel prizes, one for discovering the link between human papilloma virus and cervical cancer (by Harald zur Hausen in 2008) and another about the role of telomerase in preventing chromosome degradation (by Elizabeth Blackburn, Carol Greider, and Jack Szostak in 2011). Recently, a first detailed genomic and transcriptomic characterization of a *HeLa* cell line relative to the human reference genome (4) and an haplotype-resolved genome and epigenome of the aneuploid *HeLa* cancer cell line (5) has been published. Among the various applications based on *HeLa* cells (such as for instance for developing treatments for syphilis, AIDS, and cancer), it is worth remembering one of the earliest and important one, ie, the development of the vaccine against the polio virus (6). *HeLa* cell lines are commercially available and also circulate freely within the scientific community.

The biological characteristics of the *HeLa* cells showed to be clinically extraordinary, as described by Howard W. Jones who conducted the gynecological examinations and found that the tumor was soft, difficult to identify by the bare fingers, its color was purple and “…general examination was completely negative. Inspection of the cervix, however, revealed a lesion […] smooth, glistening, and very purple […]. Its appearance was different from any of the other 1.000 or so carcinomas of the cervix I had previously seen” (7). It also did not respond to radiotherapy. But what was amazing of this tumor, was its capability – even compared to other cancer cells – of rapid propagation and unusual invasiveness and to be durable not only inside Henrietta’s body, but also outside, in laboratory.

Nowadays, we know that the cause of the proliferation capability of these cells is related to an active version of the telomerase which, during the cell divisions, prevents the incremental shortening of the chromosome telomeres (8), which is implied in aging and eventual cell death. Because of their persistency, contamination of other cell lines by *HeLa* cells is frequent, thus they have been also referred as “laboratory weed” (3). Aneuploidy (chromosome number 82 with four copies of chromosome 12 and three copies of chromosomes 6, 8, and 17) is documented in *HeLa* cells, as result of horizontal gene transfer from human papillomavirus 18 (HPV18) to human cervical cells (9). In culture conditions, *HeLa* cells divide unlimitedly (“immortal” feature) and may mutate: hence from the same tumor cells removed from Henrietta, many strains of *HeLa* cells have been generated. Estimations of their total number spread in the laboratories and repositories all over the world give amazing quantities which far exceed the total number of cells that were in Henrietta's body (10). Worth stressing, *HeLa* cells have even been proposed to be regarded as the contemporary establishment of a new species (*Helacyton gartleri*) because of their ability to replicate indefinitely, their own clonal karyotype, their chromosomal incompatibility with humans, their ecological niche, and their ability to persist and expand (11).

The *HeLa* cells have been also presented as a paradigmatic example of fraud and prevarication of bio-ethics since neither Henrietta Lacks nor her family have been informed about the use of *HeLa* cells, and anonymity was compromised through the naming of the cell-line (12). The *HeLa* cells case became popular recently, with the publication, in 2010, of the book “The Immortal Life of Henrietta Lacks” by journalist Rebecca Skloot which was winner of several awards and became a best-seller. In the last months, moreover, the sequencing of *HeLa* genome (4,5) was object of a new important case concerning consent and privacy since, even though the genomes of the cells used in these studies are not identical to Lacks' original genome, still their sequence may reveal some heritable aspects which would violate descendants’ privacy. Because of this fact and also because of the extraordinary scientific and bioethic significance of *HeLa* cells, in August 2013, an agreement was resolved between the US National Institutes of Health (NIH) and Henrietta’s family members. Accordingly, the sequence data were placed *“in a controlled-access database*,*”* ie, the NIH's database of genotypes and phenotypes (dbGaP; *http://www.ncbi.nlm.nih.gov/gap*), “which would require researchers to apply to the NIH to use the data in a specific study and to agree to terms of use defined by a panel including members of the Lacks family.” This agreement is expected to urge new “discussions regarding consent for future use of bio-specimens, with a goal of fostering true partnerships between researchers and research participants” (13).

## From *HeLa* “cells” to the *HeLa* “bio-object”

The concepts of bio-object and bio-objectification have made possible to describe and discuss the *HeLa* cells with a consistent set of features that describe the process of how they come into being as biological phenomenon, research object, and commercial product, and how this shift is part of a more complex interaction between biology, science, technology, and society.

*HeLa* cells possess features of a bio-object which seem to be of particular relevance, first of all the potentiality to cross barriers. In Henrietta’s human body, in fact, a virus induced DNA modifications resulting in their immortalization. Thus, hybridity (14) may be suggested as an outcome of the interaction between different domains (virus/mammalian), while the ‘transformation’ of Henrietta Lacks’ cells by the virus, produced a new entity, a boundary crawler (15) between human/non-human.

Some important characteristics of these cells also concern bio-social implications we recognize to the bio-objects. Principally, this tumor raises property issues. It was part of Henrietta Lacks’ body, belonged to her because it was inside her and existed due to her, thus being her property. On the same time, however, it may be identified as something separate from her, in the form of a parasitic, invasive, and transgressive biologic material to be dissected. Outside the body, its survival became technology-dependent (from cultural media, conditions, and repositories). Separate from her, this “medical waste” became a precious material to be shared, sold, and disputed. It acquired the identity of a tool to study, but also to generate other bio-objects in a circular process where the new knowledge is the starting point of new bio-objectification leading to the production of further bio-objects.

*HeLa* cells can also be described as part of a hermeneutical process where their meaning is produced through a continuous and dynamic process of interpretation. They represent a multitude of meanings depending on context and interpretative approach, hence they require new policies and communication practices (16).

The importance of *HeLa* cells raises from the combination of some crucial factors, concerning their easy culture (fast growth, immortality, and desirable stability); their availability through large scale industrial production and distribution; their fame in the laboratories all over the world; and finally their low cost.

## Phenomenology, epidemiology, and personalized medicine

The story of Henrietta Lacks’ life is in sharp contrast to the story of the life of her cancer cells. As a poor black woman, she represents the margin of the society, the “other.” Her cancer cells on the other hand, have characteristics that make them especially valuable for the research. They are valuable because they made possible to study essential aspects of what it implies to be human. Seen in relation to the emphasis on quantitative medical research (eg, bio-bank research) in today’s society, Henrietta Lacks’ cells underline the value of qualitative research in medicine.

Qualitative method, phenomenology, and hermeneutics are well known from caring sciences like nursing sciences. With direct links or roots in Heidegger’s philosophy, this phenomenological method is developed to uncover human concerns and practices that are central for being and dwelling in the world. Focus is on experiences. As a method, it helps in identifying contextually bound clusters of themes, and it makes interpretation a key issue for scientific analysis as it is essential to understand phenomena as part of processes and context (17). The interpretative process is dynamic, and strives to go from the part to the whole, in a manner where critical reflection of the process is emphasized, aiming at achieving insights of general value.

In contrast to *HeLa* as a single cell line, nowadays in the field of molecular biology, epidemiological research on common complex diseases (CCD) is based on large databases, not the least bio-bank data. This large scale approach is high on the agendas of academic as well as public and popular debates and of regulatory work at Communitarian and national levels in Europe. In part, this reflects how new technology has changed epidemiological research in the last 10-15 years, and how ethical and legal regulations collide with medical and commercial visions in this field. Cancer investigations conducted on *HeLa* cells represent an interesting contrast with epidemiological research on CCD. This latter, in fact, focuses on groups and is closely linked to the discourse of personalized medicine. Here, large collections of samples are converted into data, combining huge demographic databases, health record databases and survey collections, ie, combining quantitative and qualitative materials.

In personalized medicine, representation and categorization are key themes, as identification of a group and its representative are crucial. In public discourses on epidemiology and personalized medicine, on the other hand, under-representation is a hot topic as medical research is considered not fully reliable for the various groups, being conducted mostly only on a restricted population (white and young men), thus neglecting the other groups. Moreover, this fact would reinforce the main social and political categories of the 20th century, namely gender, race, ethnicity, and class (18,19). Against this background, the modeling based on *HeLa* cells is politically and socially interesting and epistemologically and ontologically challenging. The scientific value of *HeLa* cells, which have been generated from a rare cancer of a poor black woman, have been applied for creating models, to be valuable not only for the specific but also for the general biology. If new knowledge can be extracted from research on Henrietta Lacks’ tissue, does that imply that unicity is an ontological premise for cancer research? Is there a hierarchical relation between unicity and variety?

The method used in cancer research based on Henrietta Lacks’ cells represents a continuous interpretative process, going from the detail to the whole and back again, in a circular critical rethinking of contexts and processes according to the phenomenological approach in caring sciences. In epidemiological research aiming at personalizing medicine and treatments, knowledge on basic biological functions, as well as possibility to test and experiment are required. The *HeLa* cells make possible both, being used as experimental model and being tools for assays, like in a hermeneutical (interpretative) circuit. Thus, from the *HeLa* cells, an exceptional phenomenon, knowledge has been gained about the general, which in turn has been applied for defining personalized medicine and treatments, which again generate new knowledge.

## Common complex diseases (CCD), epidemiology, and *HeLa* cells

The importance of cancer in global society, and the focus on epidemiological research, sheds new lights on the value of the *HeLa* cells since, according to the official World Health Organization (WHO) data, cancer is an increasing cause of death, globally (20). Besides, epidemiological research on CCD (cancer, diabetes, Alzheimer, Parkinson, and cardiovascular diseases) is a main tool for developing diagnostic, identifying causes, and individualizing treatments. Research based on *HeLa* cells is not only still important but has been even revaluated (3).

Epidemiological research on CCD is a quite noteworthy area for society, individual’s health and well-being, as well as for economy. Medical biotechnology has generated new opportunities and also loud ethical and legal debates and a new landscape of directives, regulation, and law (18,21). Concerns on the draft of EU-Data Protection Regulation has been driven by geneticists and ethicists involved in bio-bank research on CCD, the first being critical toward possible research limitations and the second being divided in pro and con fractions toward the use of huge databases of personal data (22,23). In this framework, bio-bank research appears as a main road for research breakthrough while personalized medicine is regarded as the new area for diagnostics and treatments. However, focusing this latter on group specific characteristics, doubts have raised on their effectiveness in representing populations since the chosen categories could be reproductions of established social categories instead, thus not representing the medical relevance required (19).

If the basic research developed on the *HeLa* cells may not be controversial in itself and thus does not create public controversy, when these cells also regard bio-bank issues, conversely, some quite problematic questions may rise, among them, variety and diversity. *HeLa*-cells, in fact, would represent the value of the one, while epistemological assumptions in epidemiological research on bio-bank collections would represent the value of the many. Accordingly, doubts can be raised whether fundamental biology research based on *HeLa* cells would represent and produce additional understanding of human constitution, health, and well-being considering epidemiological research and fundamental biology research.

## Toward a new paradigm of science

In the field of medical biotechnology, like in science overall, research and society are intimately related (24) and changes of one reflect the other. Policy is a relevant and often controversial outcome of this interaction, as the bio-objectification process points out (25). In the case of bio-bank research, an important context is the new EU draft concerning data protection regulation (26), as well as the establishment of the new European bio-bank network (Biobanking and Biomolecular Resource Research Infrastructure, BBMRI) (27), which concern the use of new qualitative and quantitative materials and methods, which create new challenges for researchers, clinicians, and regulators. In this context, the draft of EU data protection regulation is object of laud public debates and disputes.

Our social economic setting is organized in private and public spheres, in communal and individual ownership. The industry and ethical guidelines have so far focused on informed consent, anonymity, benefit share, property, and ownership. Regarding bio-economy in Europe, research and commercialization have been split in public and private sectors, where information about the individuals has been restricted to the public institutions. Now, the need of co-operation between medical research and pharmacy, as well as the use of personal data create new challenges regarding privacy, autonomy, and governance. Thus, a new challenge is emerging – both from society and research – concerning the most suitable models for organizing and governing the advancement of biotechnology research while on the same time protecting the citizens.

The EU data protection regulation draft aims at building up a joint platform for the EU with common rules as well as common interpretations of established rules (being this latter point a constraint up to now) and centralized control functions (26). This document pushes forward the relevance of informed consent as its key ethical concept. At the same time, it aims at facilitating unity and cooperation within the EU where materials are meant to be shared, overcoming the present national obstacles concerning laws and regulation. In addition, BBMRI (27) has already grown in to a 54-member consortium with more than 225 associated organizations (largely bio-banks) from over 30 countries, making it one of the largest research infrastructure projects in Europe. As already pointed out (28), “The BBMRI has proposed the concept of expert centers, in which pharmaceutical research would be conducted outside the industry setting, donor material would not move outside bio-bank infrastructure, and industry would not have exclusive rights to data generation.” With this platform and the draft of EU-Data Protection Directive, EU aims at strengthening the protection of the individual (informed consent) and at bringing together formerly separated public and private institutions and sectors, ie, it is starting a change regarding governance and ownership of knowledge and property. This approach is expected to make real that new vision of the role of biotechnology and bio-industry in EU and in individual nation states (29).

We believe that these changes, ie, a new perspective of knowledge production and ownership, can also be regarded as the starting point of a shift toward a new of paradigm for science where life sciences, social sciences, and humanities will jointly contribute to the human well-being.

In conclusion, in this article we have shed lights on the relation between science, society, and policy from the angle of the bio-objectification process of the *HeLa* cells. The high-quality of these cells is the result of an odd combination of their unusual features that make them a model cell for biology and medicine, with the legal, technological, and socio-economic contexts in which they have been produced, distributed, and used. As a result, they generate questions about representation, significance, and value of the single and exceptional, and the power to modify and change they can generate. Thus, as expressed with the hermeneutics terminology, they appear as a phenomenon process where new meanings and bio-realities are created. These cells disrupted and undermined the being of Henrietta Lacks, while the life of *HeLa* cells is opposed to the being of Henrietta Lacks. So, the *HeLa* cells and not Henrietta Lacks have come to represent life. Seen from the angle of present discourses on bio-bank research and data protection regulation, the bio-objectification process of the *HeLa* cells seems to be part of a further societal and political discourse on variety, individuality, and property. Belonging to one human being, *HeLa* cells have represented humanity, and emphasized the importance of individual as a core concept of the personalized medicine.
